# A prospective study of MRI biomarkers in the brain and lower limb muscles for prediction of lower limb motor recovery following stroke

**DOI:** 10.3389/fneur.2023.1229681

**Published:** 2023-10-24

**Authors:** Mat Elameer, Hannah Lumley, Sarah A. Moore, Katie Marshall, Abi Alton, Fiona E. Smith, Akif Gani, Andrew Blamire, Helen Rodgers, Christopher I. M. Price, Dipayan Mitra

**Affiliations:** ^1^Department of Neuroradiology, Royal Victoria Infirmary, Newcastle upon Tyne, United Kingdom; ^2^Stroke Research Group, Newcastle University, Newcastle upon Tyne, United Kingdom; ^3^Department of Sport, Exercise and Rehabilitation, Northumbria University, Newcastle upon Tyne, United Kingdom; ^4^Department of Medical Physics, Newcastle University, Newcastle upon Tyne, United Kingdom; ^5^Department of Neuroscience, Manchester Metropolitan University, Manchester, United Kingdom; ^6^Department of Stroke Medicine, Royal Victoria Infirmary, Newcastle upon Tyne, United Kingdom; ^7^Newcastle Magnetic Resonance Centre, Newcastle University, Newcastle upon Tyne, United Kingdom

**Keywords:** stroke, biomarkers, MRI, recovery, lower limb, strength, spectroscopy, tractography

## Abstract

The aim of this prospective observational longitudinal study was to explore and decipher the predictive value of prospective MRI biomarkers in the brain and lower limb muscles for 3-month lower limb motor recovery following stroke. In the brain, we measured the integrity of the corticospinal tract (fractional anisotropy/“FA”). In the muscles, we measured volume, fatty replacement (fat fraction analysis and proton spectroscopy) and oedema. Measurements were taken at two time points: (1) within 4 weeks of stroke (baseline measurement, clinical and imaging) and (2) 3 months following stroke (follow up measurement, clinical only). Clinical measurements consisted of assessments of functional ability and strength (Fugl-Meyer score, motor NIHSS, Functional Ambulation Category/“FAC”, and muscle dynamometry). Twenty-three patients completed imaging and clinical assessments at baseline and follow-up; five patients had partial imaging assessment. The results provided some evidence that damage to the corticospinal tract would result in less motor recovery: recovery of the Fugl-Meyer score and dynamometric ankle plantarflexion, ankle dorsiflexion, and knee extension correlated positively and significantly with fractional anisotropy (0.406–0.457; *p* = 0.034—*p* = 0.016). However, fractional anisotropy demonstrated a negative correlation with recovery of the Functional Ambulation Category (−0.359, *p* = 0.046). For the muscle imaging, significant inverse correlation was observed between vastus lateralis fat fraction vs. NIHSS recovery (−0.401, *p* = 0.04), and a strong positive correlation was observed between ratio of intra- to extra-myocellular lipid concentrations and the recovery of knee flexion (0.709, *p* = 0.007). This study supports previous literature indicating a positive correlation between the integrity of the corticospinal tract and motor recovery post-stroke, expanding the limited available literature describing this relationship specifically for the lower limb. However, recovery of functional ambulation behaved differently to other clinical recovery markers by demonstrating an inverse relationship with corticospinal tract integrity. The study also introduces some muscle imaging biomarkers as potentially valuable in the prediction of 3-month lower limb motor recovery following stroke.

## 1. Introduction

There are unique challenges posed by stroke and its associated morbidity. Current estimates place over one million stroke survivors in the UK, with societal costs estimated around £26 billion and projected to rise to over £68 billion annually by 2035 (1). Much of the societal and personal cost of stroke is due to stroke related impairments with nearly three quarters of stroke survivors report leg weakness (2). Further to loss of leg power, stroke has a significant and multifactorial effect on walking. Whilst ~85% of all people who survive a stroke can independently walk after 6 months, a large proportion (~40%) of survivors unable to walk independently early after stroke do not regain this function with time (3–5). While there are individual variations in motor recovery, most of the post-stroke recovery takes place within the first 3 months with significant slowing of recovery beyond this period in most cases (6–8).

It is important to understand and predict the motor recovery of stroke patients (9–11) to help clinicians discuss prognosis with patients and families; to help provide individualized rehabilitation (for example, focusing on recovery if likely to be successful, or on adaptation if recovery is unlikely); and to facilitate rehabilitation research by reducing bias caused by differences in initial prognosis in the study arms.

Despite the high incidence of stroke and the significant associated disability, the ability to accurately predict motor recovery following acute ischaemic stroke remains limited. Most of the literature regarding prognostic biomarkers has emphasized upper rather than lower limb recovery (10–14). Advanced brain imaging biomarkers (for example, imaging of function or white matter microstructure) have been found promising for the prediction of upper limb recovery (15–17) which, when combined with clinical markers, have the greatest prognostic accuracy (12). The exact neuroanatomical location of the infarcts can have a significant impact on motor and non-motor outcomes, with damage in the region of white matter tracts being a poor prognostic feature (18). Although the impact of persistent lower limb deficits is also significant only a small number of studies have attempted to translate these neuroimaging and clinical prognostic biomarkers to investigate lower limb motor recovery (8, 19–22), the most successful of which was the TWIST algorithm which favors clinical markers over brain imaging biomarkers (5); however, this has yet to be externally validated and internal validation (*N* = 93) found up to 17% of patients' recovery trajectories unaccounted for by the algorithm (8).

Although tracts connecting the brain and muscles are bidirectional, to date, most stroke motor recovery studies focus solely on the source organ (the brain). Few studies have investigated the target organ (muscle) which may be as important for predicting recovery.

In this study we aimed to address these gaps in the literature by exploring the relationship between lower limb motor recovery at 3 months with MRI markers of structural damage to the corticospinal tract and lower limb musculature change. Our aim was to explore the possible utility of advanced and novel MRI variables which could indicate tissue changes in brain and lower limb muscle (referred to as MRI biomarkers) associated with the recovery of stroke patients.

Specifically, we set out to test the hypotheses that:

1. Increased damage to the corticospinal tract within 4 weeks of stroke measured by lower fractional anisotropy (FA) or reduced whole-tract volume may result in less recovery of lower limb impairment and walking ability at 3 months.
2. Early features of muscle oedema, atrophy, or macro/microscopic fat redistribution may be detectable within 4 weeks after stroke and may correlate with impaired recovery of lower limb power and walking ability at 3 months, and/or with markers of increased damage to the corticospinal tract.
3. Combining brain and lower limb biomarkers in a regression model may result in more accurate predictions of motor recovery than considering individual biomarkers alone.

## 2. Methodology

### 2.1. Study design and setting

This study was designed as a prospective observational longitudinal cohort study, consisting of two study time points: the first timepoint within 4 weeks of stroke onset consisting of both imaging and clinical measurements; and the second timepoint at 3 months after stroke onset consisting of clinical measurements only. The study was designed so that the brain and lower limb MRI assessments were performed blinded to each other and to the clinical details.

Recruitment started in October 2019, and the last patient completed follow-up in March 2022. Participants were enrolled from five stroke units across two major healthcare trusts in North East England. The clinical assessments were performed within hospital, at the participant's home, or at the Newcastle Magnetic Resonance Center at Newcastle University. Research MRI scans were also performed at the Newcastle Magnetic Resonance Center.

### 2.2. Participants

For inclusion in the study, patients had to be ≥18 years old and within 4 weeks of their first ever unilateral, suspected supratentorial ischaemic stroke causing a unilateral lower limb motor deficit (± an upper limb motor deficit) as defined by a Medical Research Council (MRC) strength score at least >1 and at best <5.

Patients were excluded if they had: (1) An absolute contraindication to MRI (e.g., pacemaker and metal implants). (2) Suspected posterior circulation or primary hemorrhagic stroke. (3) Previous history of anterior circulation stroke (clinical or radiological evidence) or posterior circulation stroke with residual clinical deficit. (4) A lack of capacity to provide informed consent to participate. (5) An inability to answer the MRI safety questionnaire. (6) Moderate to high level of pre-stroke dependency (modified Rankin Score > 2). (7) Any other pre-existing comorbidity causing a significant lower limb deficit. (8) Inability to transfer with the assistance of one or two people, depending on the recruitment site. (9) Inability to attend the 3-month follow-up assessment.

### 2.3. Clinical assessment procedures and variables

Potentially eligible participants were identified by the study PIs within each organization (AG/SM), and an appropriately trained research assistant (HL/AA) also actively screened new admissions to the participating stroke units. Once identified, a two-step screening and consent process was performed as approved by an NHS research ethics committee.

**Figure 1:**
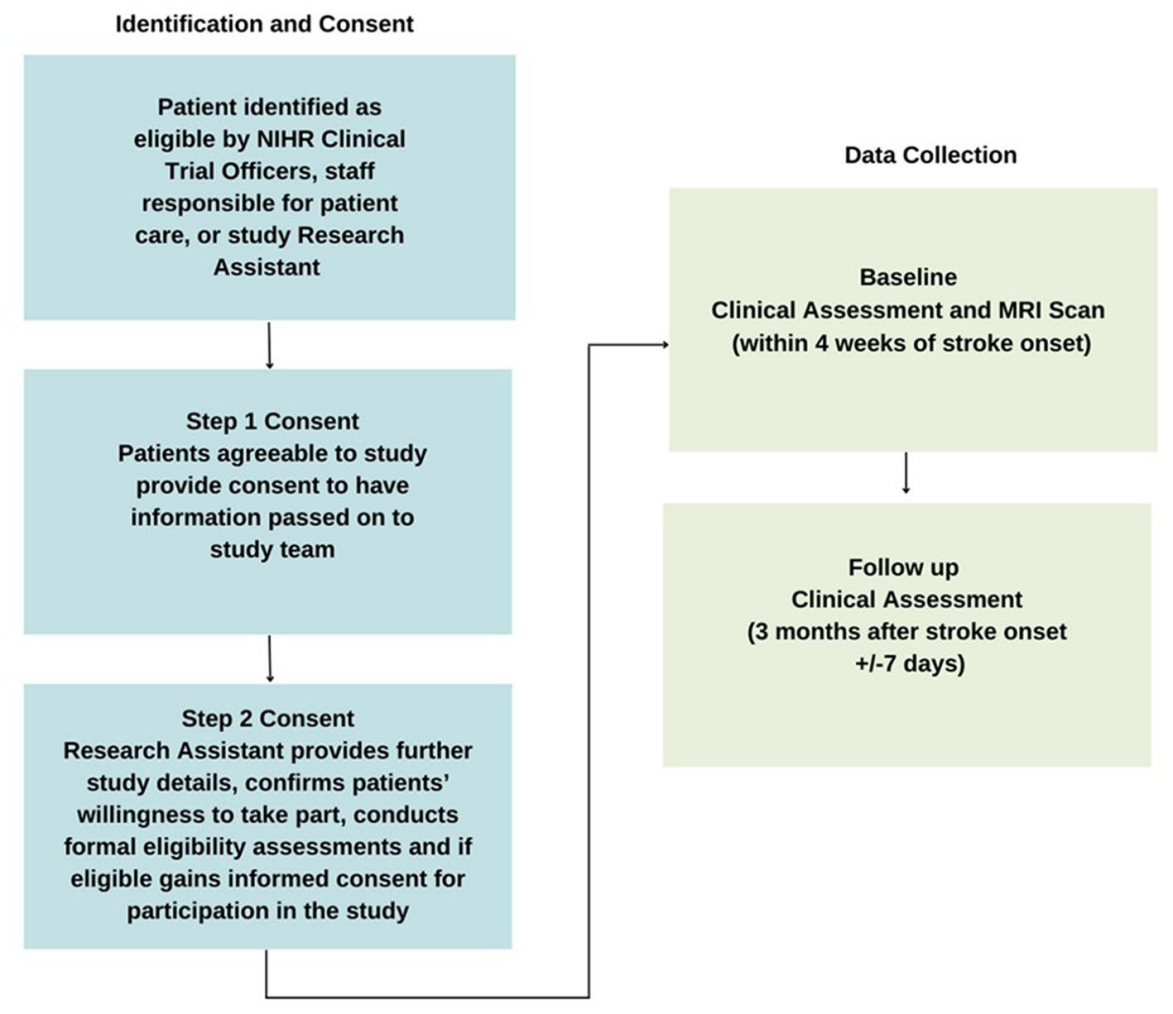
Consent and data collection procedures.

The consent and data collection procedures are detailed in Figure 1.

Baseline demographic and clinical data were recorded by the research assistant, including patient demographics (age, sex; self-reported handedness), date of stroke onset, pre-morbid modified Rankin score, pre-morbid walking status (independent or not), associated co-morbidities, e.g., presence of diabetes, hypertension, arthritis of affected limb, and stroke treatment (including thrombolysis/thrombectomy). Leg dominance is complex and task specific but generally follows hand dominance (23)—given the practical difficulties in objective leg dominance assessment for post-onset stroke patients, we extrapolated leg dominance from self-reported hand dominance.

Baseline and follow-up clinical assessments included measurements of lower limb impairment [Fugl-Meyer Lower Extremity score (FM), lower limb motor National Institutes of Health Stroke Scale (NIHSS), and hand-held dynamometry measurement], and lower limb activity [Functional Ambulation Category (FAC)].

The Fugl-Meyer score was selected as a well validated tool to assess the volitional movement, reflex activity, coordination and speed of movements around the ankle, knee, and hip (24–26). The lower limb motor component of the NIHSS provides a complementary assessment of lower limb power by testing the ability to raise and hold the leg against gravity (26, 27). As both these scores provide categorical assessments, we also included hand-held dynamometry as a continuous quantitative measurement of lower limb motor strength (28) for flexion and extension of the ankle, knee, and hip, in addition to abduction and abduction of the hip. The Functional Ambulation Category (FAC) provides a categorical assessment of the ability to walk independently (29). Further detail can be found on these clinical assessments in Appendices 1–4.

Fugl-Meyer and NIHSS scores were reported for the affected limb, and the dynamometry was normalized by calculating the ratio of values from affected:unaffected limbs to correct for any baseline differences. Recovery was defined by the value of dynamometry, FAC and FM at follow-up minus baseline, and baseline minus follow-up for NIHSS.

All assessments were performed following dedicated training and were indirectly supervised by a Clinical Professor of Stroke Medicine (CP) and an Assistant Professor of Sport, Exercise, and Rehabilitation (SM).

### 2.4. Imaging assessment procedures and variables

All MRI scans were performed on a Philips Intera Achieva 3T system (Philips Healthcare, Amsterdam, Netherlands) at the Newcastle Magnetic Resonance Center.

#### 2.4.1. Lower limb imaging

For MR data acquisition the body radiofrequency coil for signal transmission and surface-array coils for signal transmission were used. Patients were positioned feet-first supine and lower limbs were scanned bilaterally from the greater trochanter to the knee joint. The core protocol consisted of T1-weighted, Short Tau Inversion Recovery (STIR), 2D, 3-point Dixon, and a single-voxel Point RESolved Spectroscopy (PRESS) sequence with 96 signal averages performed at rest with voxels placed in the vastus lateralis muscles bilaterally. MR acquisition details are given in Appendix 5.

#### 2.4.2. Brain imaging

Whole-brain structural imaging was obtained using a volumetric T1-weighted sequence and 3mm thick axial spin-echo T2 slices covering the whole brain. A 64-directional (*b* = 1,000 s.mm^−2^) diffusion weighted acquisition was acquired including 6 acquisitions with no diffusion weighting, and an identical non-diffusion weighted image was acquired with reversed phase encoding gradients for distortion correction. The directional diffusion of water was used as a surrogate marker for white matter tract mapping and integrity (30). Specifically, the fractional anisotropy (FA) and calculated tract volumes were extracted as key biomarkers—we used FA because it contains information about damage to white matter tracts more specifically than MD, which contains information about damage to other kinds of brain parenchyma. This is because MD averages away the differentiation in diffusion signal over multiple directions, as opposed to FA which specifically measures the unidirectional preference of diffusion signal within a voxel. Further, we used FA rather than separately describing axial and radial diffusivities (AD and RD) because it is the most complete and widely reported single tractography biomarker, is sensitive to changes in stroke patients, and integrates information about both axial and radial diffusivities (30, 31). Further detail can be found in Appendix 5.

#### 2.4.3. MRI and MRS data processing

Leg and brain imaging were separated to prevent observer bias, and all analyses were conducted blinded to the patient's clinical status. For all leg and brain MRI biomarkers, the affected side was normalized against the unaffected side to account for background variability.

Vastus lateralis muscle quality was assessed on T1-weighted imaging using the 6-point semi quantitative scale (32, 33) proposed by Mercuri et al. and STIR imaging was assessed to indicate the degree of macroscopic oedema using a 4-point qualitative scale (no change, mild, moderate, or severe increased signal). These were both performed by two consultant diagnostic neuroradiologists (ME and DM).

All quantitative MRI lower leg data were processed using in-house written software in Matlab (MathWorks, Natick, MA, USA). Dixon data were reconstructed (34) using a six-component lipid model and considering a single T2^*^ decay. Fat fraction (FF) was calculated as:


$$Fat\;fraction=\frac{SI\;\left(fat\right)}{SI\;\left(fat\right)+SI\;\left(water\right)}*100$$

**Figure 2:**
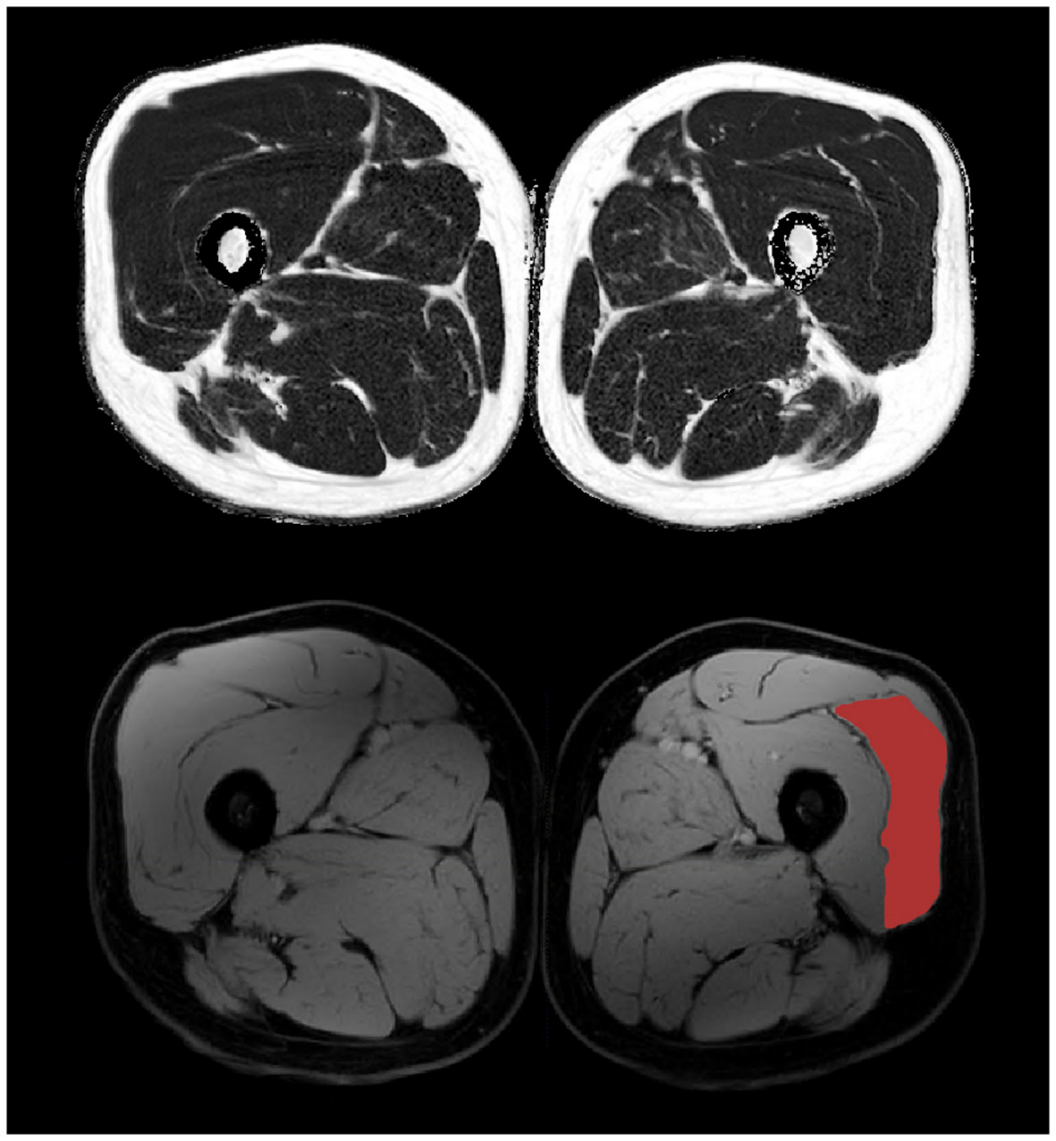
3-point Dixon mid-thigh MRI data with reconstructed fat **(top)** and water **(bottom)** maps. The vastus lateralis muscle is highlighted on the bottom right.

SI = signal intensity. FF maps that included subcutaneous and bone FF values < 95%, or partial fat-water swaps would have been excluded for analysis (none met this threshold for exclusion). Reconstructed Dixon data is shown in Figure 2. A three point Dixon technique is particularly robust for calculating quantitative fat maps, yet is also a fairly quick and easily reproducible MRI sequence. The algorithms used rely on measuring the amount of dephasing caused by chemical shift from intra-voxel fat and water mixing (the resonance frequency of protons in the fat and water environments are slightly different) (35). The advantage over more specific spectroscopy analysis as described below is the ability to cover much larger areas of muscle with good spatial resolution within a practical time frame.

Regions of interest (ROIs) were drawn manually on the water image by a clinical medical physics post-graduate student (KM) using the free software tool (www.itksnap.org). The Vastus Lateralis muscle was delineated bilaterally avoiding inclusion of other muscles, subcutaneous and intermuscular fat, tendons and major blood vessels for five central slices (150 mm of the muscle length) centered at maximal muscle thickness. FF for an average of these segmented volumes areas were calculated from the registered fat-fraction map in ITK-SNAP, as was the volume of segmented contractile muscle.

**Figure 3:**
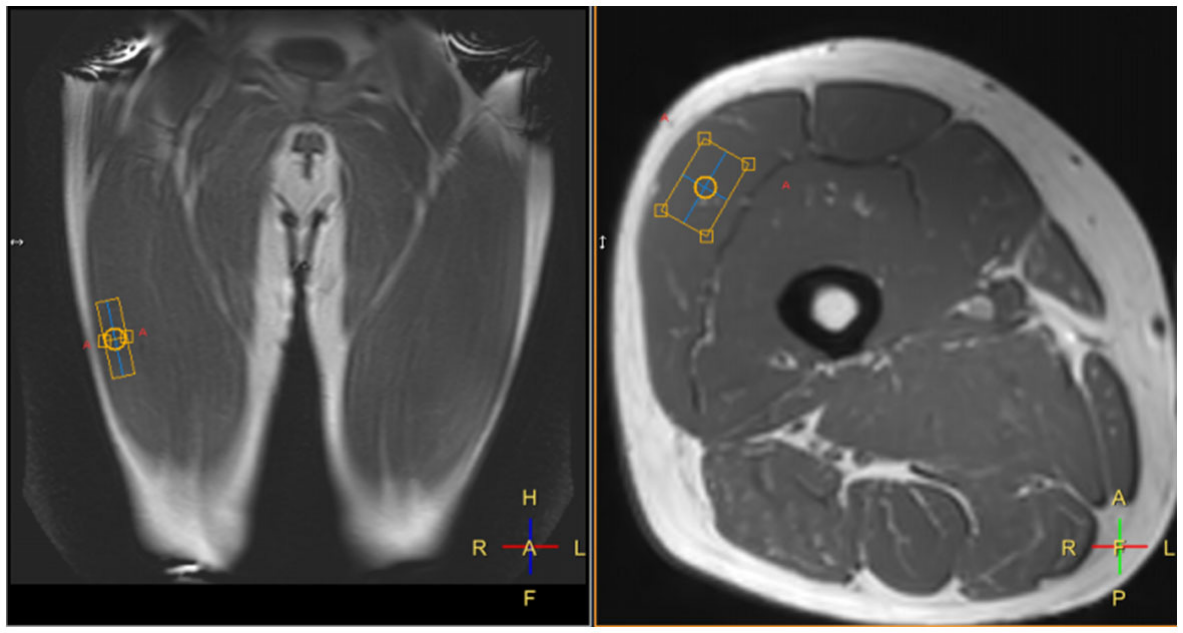
Example placement of a spectroscopy voxel (orange box) within the right vastus lateralis muscle.

**Figure 4:**
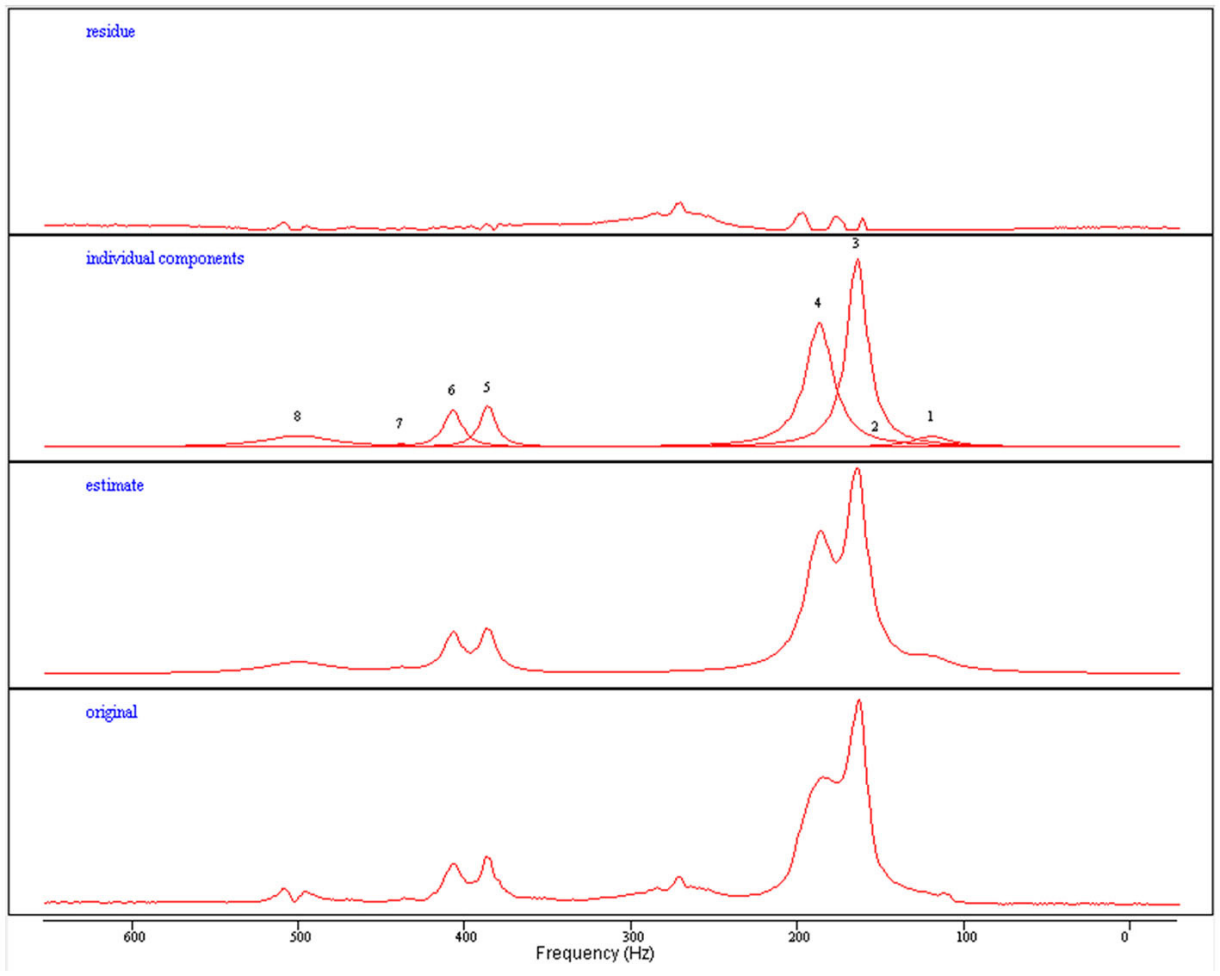
Quantification process for extracting intra- and extra-myocellular lipid concentrations from acquired proton spectroscopy. Original: Raw spectroscopy data. Estimate: A model fitted to the generated data. Individual components: Individual peaks can be de-convolved to separate from each other (3—IMCL, 4—EMCL). Residue: Represents the deviation from the raw data.

Spectroscopy analysis was performed by an experienced MR physicist (FES). Spectroscopy can very accurately delineate the concentration of molecules with distinct resonance frequencies (36), with the trade-off that in order to distinguish peaks with similar resonance frequencies, a high signal to noise ratio is required—which can be obtained by utilizing a larger voxel size, but at the expense of spatial resolution. This technique enables not only the measurement of fat concentration within muscles, but also in many cases the intra- and extra-cellular lipid concentrations can be differentiated (35). Spectroscopy is widely used in clinical neuroradiology practice to investigate brain tumors and other diseases (36), and has been used in the research setting to investigate muscle composition (35, 37). Example voxel placement can be seen in Figure 3. All spectra were analyzed using “Java-based magnetic resonance user interface” software (jMRUI version 3·0; http://www.mrui.uab.es/mrui/) and the AMARES algorithm. Signal amplitudes from intra- and extramyocellular lipids (IMCL and EMCL) were separated by peak fitting (see Figure 4) and quantified by comparison to the water proton signal from non-water suppressed spectra. Absolute and creatine-normalized IMCL and EMCL concentrations were extracted.

#### 2.4.4. Manual CST analysis

Several pre-processing steps were taken prior to analysis of the brain MRI data, which are described in further detail in Appendix 5. These included conversion from DICOM to NIFTI-1 using dcm2niix, followed by correction of eddy current and motion artifact in the diffusion volumes through use of the FSL TOPUP/EDDY algorithm.

**Figure 5:**
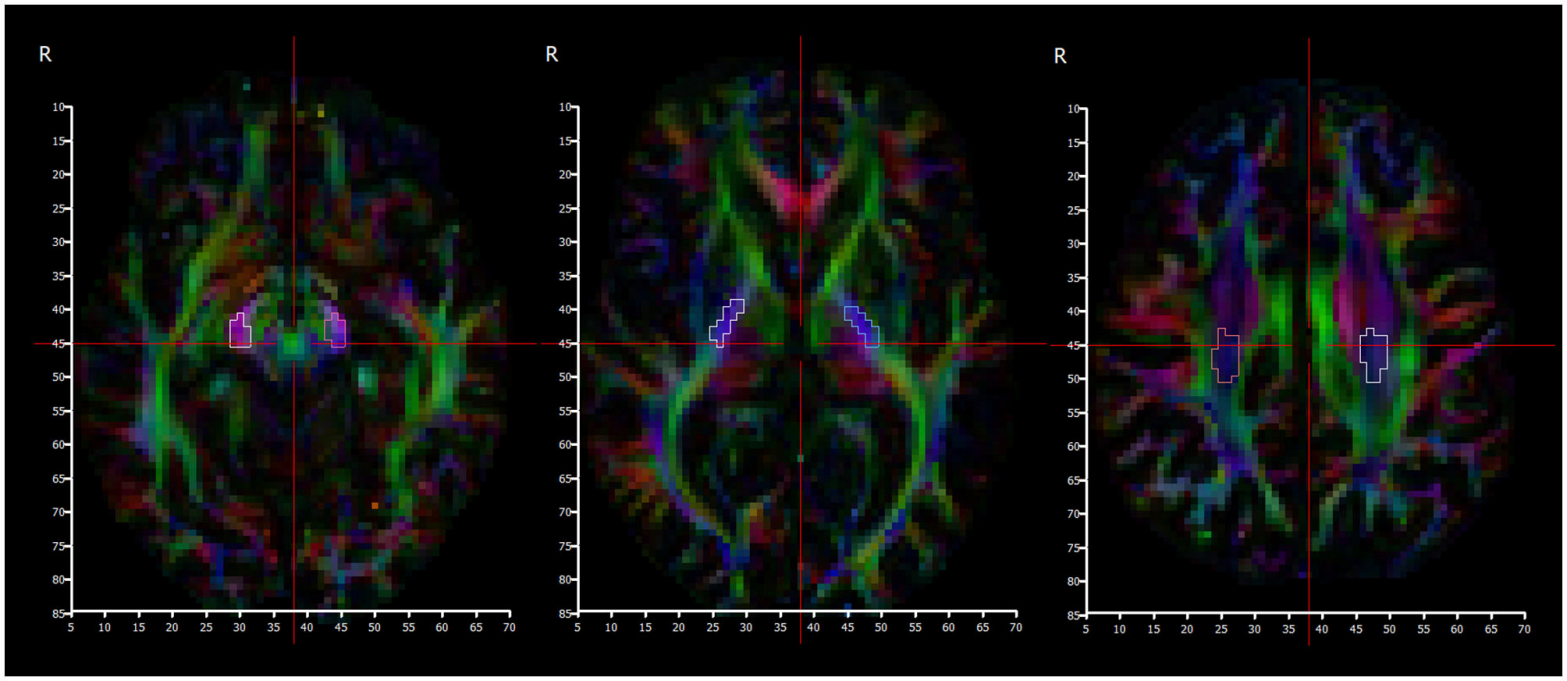
Color fractional anisotropy maps used to segment cerebral peduncles **(left)**, posterior limb of internal capsule **(middle)**, and centrum semiovale **(right)** regions.

Manual segmentation of regions of interest corresponding to the location of the corticospinal tract (CST) was performed bilaterally in the: corona radiata, posterior limb of internal capsule (PLIC), and cerebral peduncles. The corona radiata slice was selected to be one slice above the top of the ventricles; the PLIC slice was selected at the level of the foramina of Munro; and the cerebral peduncle slice was selected one slice below the bottom of the thalami (Figure 5). The volumetric T1 scan and the color FA maps were used as guidance to identify the corticospinal tract at these levels, with reference to a detailed white matter atlas (38).

Descriptive statistics were calculated in DSI studio and data describing the fractional anisotropy were obtained from the cross-sectional regions of interest corresponding to the tract at these three separate locations. The affected side data was normalized against the unaffected side to account for any background variation.

#### 2.4.5. Automated CST analysis

**Figure 6:**
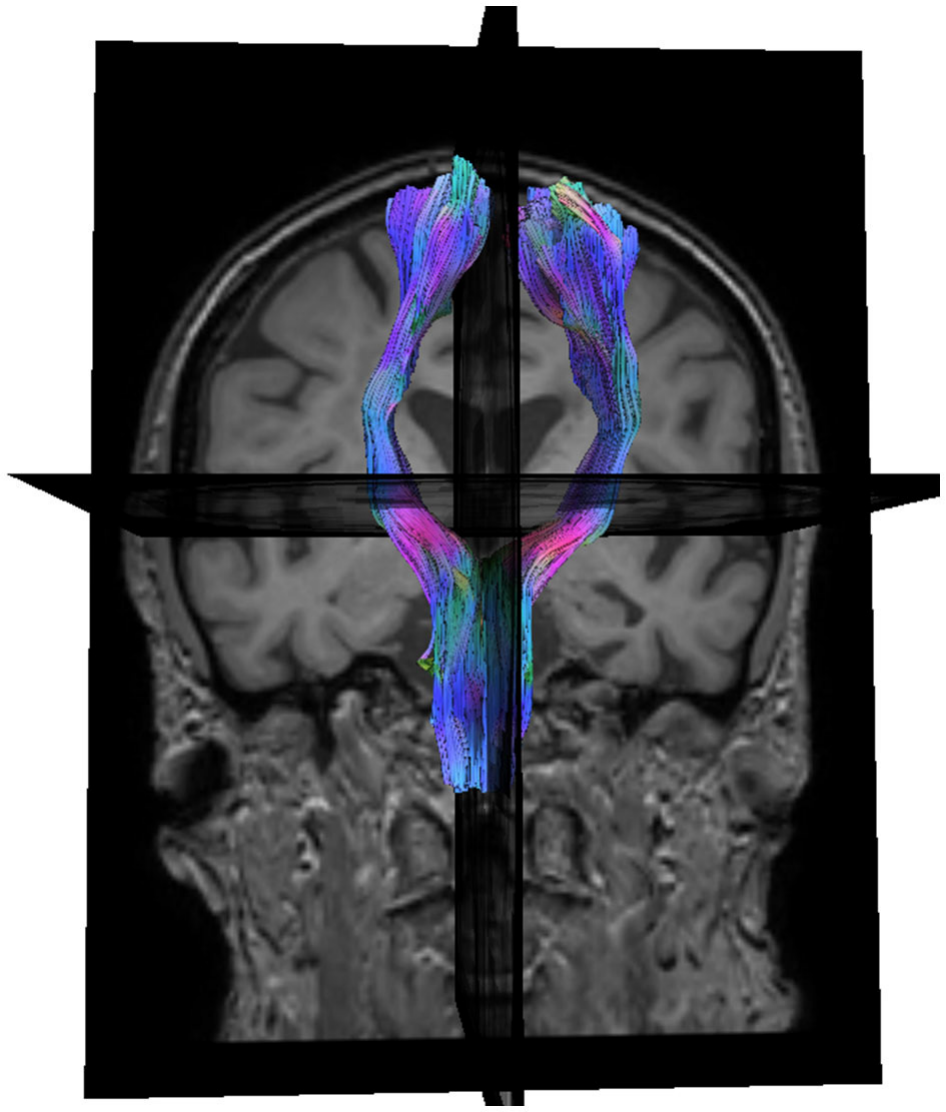
Coronal view of automatically reconstructed corticospinal tracts, rendered in 3D.

Automatic CST analysis was performed with manual supervision in DSI Studio (39–42). An example can be seen in Figure 6. The whole tract volume and average FA were extracted on either side, and the affected side normalized against the unaffected side. Further detail is provided in Appendix 5.

### 2.5. Statistical analysis

To test the hypothesis that increased damage to the corticospinal tract measured by lower FA may result in less recovery of lower limb power and walking ability after 3 months, we performed individual correlations between all DTI-derived metrics and all clinical recovery metrics, as defined in the previous section.

To test whether early features of muscle oedema, muscle atrophy (volume, fat fraction, and increased Mercuri score) or microscopic fat redistribution (intra and extramyocellular lipid concentrations) correlated with impaired recovery of lower limb power and walking ability at 3 months, we also performed individual correlations between these MRI muscle variables and all clinical recovery metrics.

Finally, the interaction between brain and lower limb biomarkers was determined by performing correlations between each brain and lower limb biomarker.

Statistical analysis was performed in SPSS. Spearman's rho was calculated with 95% *p*-values for all biomarkers to account for non-parametric and ordinal biomarkers within correlating pairs. One-sided testing was performed because our hypotheses included expected directions of correlation. All MRI biomarkers were also entered to a multivariate regression analysis as predictors of motor outcome at 3 months (lower limb Fugl-Meyer score).

As this was an exploratory study, an a-priori sample size calculation was not performed. When patients were unable to tolerate/complete the MRI scan, participants were included in any correlations for which biomarker pairs were available and automatically excluded when one or both biomarkers in a pair were not available.

## 3. Results

**Figure 7:**
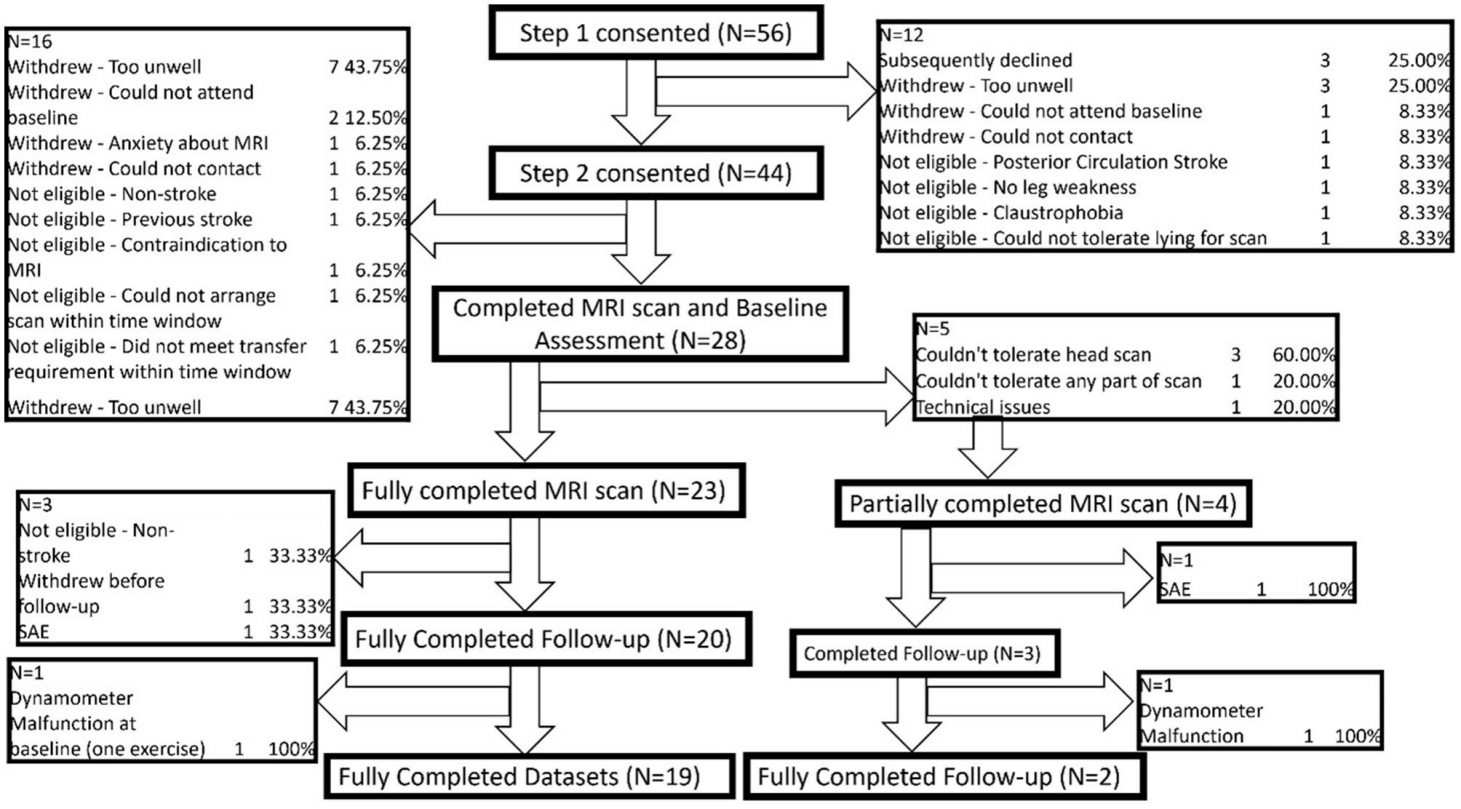
Attrition flowchart.

A cohort of 44 acute ischaemic stroke patients (mean age: 57; 26 Male, 18 Female) were recruited at Step 2. Due to participant attrition (reasons detailed in Figure 7) a total of 28 participants attended the baseline clinical assessment and MRI scan. A summary of their demographics and baseline scoring is presented in Table 1. No patients were required to stay on an intensive care unit.

**Table 1:** Individual demographic and baseline clinical data.

	**Gender**	**Age (years)**	**Affected leg**	**Dominant leg**	**Infarct location**	**Lower limb motor NIHSS**	**Lower limb MRC power**	**Baseline functional ambulation category**	**Baseline lower limb Fugl-Meyer**	**No. days post-stroke**
BA1	Male	63	Right	Right	Basal ganglia	2	4	2	29	17
BC2	Male	53	Right	Right	Frontal	1	4	5	31	8
BC3	Female	61	Left	Right	-	1	4	5	32	9
BC4	Female	81	Right	Right	Brainstem	2	4	2	26	20
BC5	Female	60	Right	Right	Basal ganglia	1	1	5	33	16
AA2	Male	62	Left	Right	Parietal	2	5	2	21	14
AA3	Male	43	Left	Left	Frontal	1	4	5	33	10
AA5	Female	77	Left	Right	Frontal	1	4	3	34	21
BA5	Male	48	Left	Left	Parietal	1	3	2	28	15
AA7	Male	67	Left	Right	Frontal	1	5	5	34	28
AA9	Male	54	Right	Right	Frontal	1	2	2	20	2
AA12	Female	66	Left	Right	Brainstem	1	4	4	32	3
AA14	Male	64	Right	Right	Basal ganglia	1	5	5	30	11
AA16	Female	55	Right	Right	Parietal	1	4	5	29	25
AA17	Male	79	Left	Right	Brainstem	0	4	5	31	22
BC8	Male	59	Right	Right	Parietal	1	4	4	34	19
BC9	Male	49	Right	Right	Brainstem	1	4	5	32	27
BC10	Male	59	Left	Right	Frontal	1	4	5	34	16
BA6	Male	68	Right	Right	Basal ganglia	1	4	5	32	23
BC14	Male	78	Left	Right	Frontal	0	4	5	33	26
BA7	Male	80	Left	Right	Brainstem	1	4	3	31	27
AA21	Male	57	Right	Right	Frontal	1	4	5	33	6
AA22	Male	48	Left	Right	Basal ganglia	2	4	5	33	11
BC16	Female	82	Right	Right	Brainstem	2	4	5	33	25
BC17	Female	71	Left	Right	Basal ganglia	2	0	4	25	27
AA24	Female	84	Left	Right	Brainstem	0	4	4	32	19
AA26	Male	42	Right	Right	Temporal	1	4	5	32	16
AA28	Male	62	Left	Right	No infarct	1	4	5	33	16

Twenty-three participants fully completed the baseline clinical assessment and MRI, and 5 partially completed due to either claustrophobia in the scanner (*N* = 4) or dynamometer failure (*N* = 1). All MRI scans were performed within 28 days of stroke onset (mean 17.2 days; range 2–27).

Of the 24 patients for whom brain MRI was completed, the infarcts were located as follows: 8 frontal, 4 parietal, 1 temporal, 6 basal ganglia, 6 brainstem. The brainstem infarcts were included because they were all suspected to be anterior circulation infarcts at the point of recruitment. One patient was excluded as they were identified as non-stroke (meningioma). Eleven infarcts were localized within the left hemisphere, and all except one patient were right leg dominant. The dominant leg was affected clinically in 13/24 (54%) cases.

14/24 (58%) leg MRIs demonstrated spectra of sufficiently quality to estimate microscopic lipid concentration, with 10/24 (42%) cases unable to resolve IMCL/EMCL peaks on one or both sides.

Two participants had significant artifacts on Dixon sequences which precluded segmentation of the vastus lateralis muscles. Two further participants (BA1 and AA2) also had outlying fat fractions felt to be uniform scaling errors after inspection were included because both limbs were equally affected so our reported variable of affected:unaffected limb ratio would normalize the discrepancy. Satisfactory quality was achieved for all other cases in the structural leg and brain imaging. Interslice agreement for the diffusion data was 83% (range: 70–86%) and all scans passed visual inspection following correction with TOPUP/EDDY.

Due to participant attrition following the baseline clinical assessment and MRI scan (reasons detailed in Figure 5) a total of 24 participants completed the follow up clinical assessment. Of these, 23 participants fully completed the follow up assessment and 1 partially completed due to dynamometer failure.

### 3.1. Statistical analyses

#### 3.1.1. Does increased damage to the corticospinal tract as measured by lower FA correlate with less recovery of lower limb motor impairment and walking ability by 3 months?

We found evidence to support this hypothesis. The biomarker data is shown in Table 2. Clinical recovery data and full results from the statistical analysis are available in the Supplementary material (Worksheets 4 and 5).

**Table 2:** Per participant brain biomarker results, reported separately for the unaffected (left) and affected (right) hemispheres.

	**Unaffected hemisphere**					**Affected hemisphere**				
	**Cerebral peduncle FA**	**Posterior limb of internal capsule FA**	**Centrum semiovale FA**	**Whole corticospinal tract FA**	**Whole corticospinal tract volume (mm** ^3^ **)**	**Cerebral peduncle FA**	**Posterior limb of internal capsule FA**	**Centrum semiovale FA**	**Whole corticospinal tract FA**	**Whole corticospinal tract volume (mm** ^3^ **)**
BA1	0.69	0.63	0.43	0.53	15,874.5	0.64	0.49	0.29	0.39	14,141.7
BC2	0.66	0.64	0.33	0.5	19,252.2	0.69	0.67	0.34	0.53	8,213.56
BC3	-	-	-	-	-	-	-	-	-	-
BC4	0.62	0.6	0.28	0.47	16,470.5	0.65	0.62	0.32	0.44	18,571.7
BC5	0.62	0.58	0.29	0.49	11,586.5	0.67	0.61	0.33	0.5	17,566.3
AA2	0.74	0.69	0.43	0.55	20,450	0.59	0.56	0.39	0.46	9,958.11
AA3	-	-	-	-	-	-	-	-	-	-
AA5	0.69	0.68	0.59	0.55	14,106.5	0.54	0.45	0.57	0.49	9,461.84
BA5	0.75	0.71	0.34	0.52	20,234.2	0.63	0.53	0.36	0.45	20,927.5
AA7	0.76	0.7	0.42	0.6	9,031.28	0.57	0.53	0.37	0.51	18,567
AA9	0.6	0.6	0.32	0.51	7,708.9	0.66	0.66	0.35	0.47	2,939.2
AA12	0.73	0.6	0.39	0.47	6,535.11	0.7	0.55	0.36	0.43	2,594.88
AA14	0.71	0.53	0.26	0.42	7,272.28	0.59	0.48	0.36	0.43	10,055.3
AA16	-	-	-	-	-	-	-	-	-	-
AA17	0.68	0.6	0.31	0.51	13,366.2	0.58	0.61	0.28	0.48	7,752.49
BC8	0.63	0.48	0.31	0.4	8,238.2	0.66	0.55	0.28	0.41	8,634.74
BC9	0.71	0.71	0.38	0.47	10,026.2	0.74	0.72	0.4	0.49	16,343.8
BC10	0.58	0.62	0.45	0.5	14,369.3	0.4	0.41	0.32	0.37	12,118
BA6	0.59	0.53	0.33	0.46	6,565.22	0.65	0.64	0.31	0.52	6,615.66
BC14	0.62	0.6	0.4	0.5	11,897.4	0.71	0.64	0.36	0.51	15,708
BA7	0.7	0.64	0.37	0.49	24,362.6	0.69	0.59	0.29	0.45	26,798.2
AA21	0.73	0.66	0.31	0.49	15,452.2	0.73	0.64	0.3	0.45	17,330.5
AA22	0.76	0.67	0.34	0.52	16,295.7	0.66	0.5	0.29	0.48	7,168.24
BC16	0.6	0.59	0.29	0.5	16,958.6	0.59	0.59	0.28	0.46	15,395.9
BC17	0.67	0.7	0.35	0.52	20,164.9	0.61	0.57	0.29	0.45	15,587.1
AA24	0.74	0.63	0.41	0.51	17,925.7	0.61	0.55	0.35	0.48	13,409.9
AA26	0.63	0.67	0.36	0.46	20,349.1	0.66	0.72	0.36	0.49	19,254.5
Mean	0.67542	0.6275	0.362083	0.4975	14,353.89	0.634167	0.578333	0.339583	0.464167	13,129.76
Std dev	0.05737	0.059178	0.070058	0.040748	5,050.671	0.070411	0.078138	0.059964	0.039149	5,813.014

Confirmatory positive correlations were observed between centrum semiovale FA and recovery of ankle plantarflexion (0.457, *p* = 0.019) and ankle dorsiflexion (0.406, *p* = 0.034). Further significant correlation was identified between cerebral peduncle FA and recovery of knee extension (0.423, *p* = 0.032).

Further support for the hypothesis was found in the recovery of Fugl-Meyer score; normalized FA measured in the centrum semiovale positively correlated with recovery of FM (0.457, *p* = 0.016).

However, we did find that the recovery of functional ambulation (FAC) contradicted our hypothesis by significantly negatively correlating with whole CST FA (−0.359, *p* = 0.046).

#### 3.1.2. Do markers of increased atrophy, oedema, or microscopic fat redistribution correlate with less recovery of lower limb motor faculty by 3 months?

We found some evidence to support this hypothesis. Per participant data from the lower limb biomarkers and clinical recovery metrics plus full results from the statistical analysis are available in the Supplementary material (Worksheets 3, 4, and 6, respectively).

We identified negative correlations between Mercuri score and recovery of hip extension (−0.386, *p* = 0.042), and NIHSS and vastus lateralis mean fat fraction (−0.401, *p* = 0.04).

We also found a negative correlation between extramyocellular lipid concentration and recovery of knee flexion (−0.591, *p* = 0.028). When the ratio of intra- to extra-myocellular lipid concentrations was calculated, this was found to correlate with recovery of knee flexion strongly positively (0.709, *p* = 0.007).

However, we did again find that recovery of FAC contradicted our hypothesis by demonstrating a positive correlation with vastus lateralis mean fat fraction (0.377, *p* = 0.046).

We also found a negative correlation between recovery of ankle dorsiflexion and vastus lateralis volume (−0.456, *p* = 0.022) that was contradictory to the hypothesis.

##### 3.1.2.1. Is there any correlation between MRI markers of increased injury to the corticospinal tract and MRI markers of oedema or fatty atrophy within the vastus lateralis muscle?

We found some evidence to support this hypothesis. The brain biomarkers are reported in Table 2, and the lower limb biomarkers along with full results from the statistical analysis are available in the Supplementary material (Worksheets 3 and 7, respectively).

When the normalized brain markers were compared to the normalized leg markers, we identified positive correlations between the vastus lateralis volume and FA measured at the CSO (0.426, *p* = 0.027), PLIC (0.599, *p* = 0.002), and CP (0.545, *p* = 0.005), in addition to the whole tract FA (0.578, *p* = 0.003) and volume (0.0416, *p* = 0.030).

Further support was found through strong negative correlation between whole tract volume and vastus lateralis fat fraction (−0.556, *p* = 0.004).

Extramyocellular lipid concentration was seen to positively correlate with FA measured at the CSO (0.577, *p* = 0.019) and CP (0.484, *p* = 0.047) which initially seems contradictory to the hypothesis, however the ratio of IMCL to EMCL demonstrated a very strong negative correlation with whole tract volume (−0.797, *p* = 0.001).

#### 3.1.3. Was there any benefit to combining MRI brain and lower limb biomarkers in a model to predict recovery?

Statistical modeling utilizing regression (automatic linear modeling for dynamometry recovery variables, and ordinal modeling for NIHSS/FAC/FM recovery variables) did not identify statistically significant predictive benefit to combining MRI variables within our small sample size.

## 4. Discussion

Damage to the corticospinal tract measured by reduction in fractional anisotropy has been linked to impaired movement following stroke in the upper limb, but there are limited studies assessing the effect on lower limb. We found several significant correlations between markers of preserved CST integrity and improved recovery of lower limb motor strength following stroke which support this hypothesis—particularly in the dynamometry and Fugl Meyer assessments. These findings support the importance of the corticospinal tract in motor control of the lower limbs and contribute to the evidence supporting the use of diffusion tensor MRI methods to evaluate the integrity of these tracts.

Walking is however more complicated than cortical motor control and power of the lower limbs: it also relies on extrapyramidal tracts such as the vestibulospinal tract and reflexes/pattern generators in the spine. Descending corticospinal tract neurons can play an inhibitory role in the modulation of these processes (43, 44), which may provide a possible explanation for recovery of functional ambulation demonstrating a significant inverse correlation with CST integrity.

One of the novel aspects to this study was the collection of MRI biomarkers of the structural integrity of the target organ (lower limb musculature). Changes in muscle structure and function, such as decreased protein synthesis, are detectable within days of a period of inactivity even in healthy adults (45) and even more so in acutely unwell patients with stroke or other conditions (46, 47). Furthermore, changes in the number of motor units of stroke patients have been shown to be reduced as early as 4 h after stroke onset. Advances in MRI imaging of muscles have enabled measurements of both macroscopic and microscopic structure to be accurately performed using widely available clinical scanners (34, 48). Combining muscle biomarkers with brain and clinical information may improve the accuracy of prognostic models for stroke recovery, and it may also provide novel insights into the neurophysiological mechanisms underlying post-stroke clinical syndromes.

Most of our patients were scanned in the subacute period after stroke and there is some evidence to suggest that at least at the microscopic level there may be structural changes identified within muscle in the subacute phase following insult (45, 49, 50). This provided the basis for our hypothesis that if we were to see any significant relationship between lower limb MRI biomarkers and recovery post-stroke, there may be early changes of atrophy (smaller volume compared to baseline or contralateral limb, and lipid infiltration) which may indicate an impaired degree of recovery.

We did find some evidence to support this hypothesis, through negative correlations between NIHSS recovery and VL fat fraction, and Mercuri score with hip extension recovery. Recovery of functional ambulation behaved in the opposite direction to what we expected, as it did when correlated with CST integrity. The strength of correlation was generally fairly weak and not consistent for all MRI and recovery biomarkers. The statistical limitations of the study are discussed below.

The MRI spectroscopy results were also of interest. Greater recovery of knee flexion was associated with a modest reduction in EMCL, but a strong increase in the IMCL/EMCL ratio. An inverse relationship was observed in the brain vs. muscle correlation—higher FAs were associated with modest increase in EMCL, but a strong reduction in IMCL/EMCL ratio. These results may suggest the importance of shift of lipid between the intra and extramyocellular lipid compartments post-stroke, of more significance than changes in the absolute concentration of either IMCL or EMCL. However, the fact that IMCL/EMCL ratio appears to reduce with higher FA yet increase with greater recovery, when the literature and our results also support higher FA being associated with greater recovery, is difficult to rationalize.

Indeed, it is important to consider the results obtained within the context of the study limitations. This was an exploratory study aiming to provide a broad assessment of the interaction between leg/brain MRI and clinical recovery biomarkers for stroke patients. As such we have evaluated many biomarkers in a relatively small sample size. Furthermore, as it was not appropriate to transport any patients with a moderate-to-severe lower limb deficit via taxi from hospital to our research scanning center (NMRC) due to medical instability, only mildly affected patients without much potential for recovery could be recruited. We attempted to navigate this problem by translating the leg MRI protocol to one of the hospital's MRI scanners (Siemens Skyra Fit 3T; Siemens Healthineers, Erlangen, Germany) to limit the need for participants to travel. This attempt, however, was unsuccessful due to hardware limitations of the scanner relating to how lateral the spectroscopy voxels can be placed. As a result of the limited available patient population, it would be difficult to generalize the findings from this study alone to the broader stroke population including patients with greater stroke severities and patients with hemorrhage.

Furthermore, despite our intention to keep the stroke onset to scan time narrow, due to local constraints in access to an appropriate MRI scanner and slow initial pace of recruitment, the recruitment window had to be expanded to up to 4 weeks post-stroke. We acknowledge that this may have led to limitations in the interpretability of our data. As presented in Table 1, most of our participants were imaged between 2 and 4 weeks after onset of symptoms.

The spectroscopy biomarkers provided further challenges in that in this patient population we were unable to consistently resolve peaks for intra- and extra-myocellular lipid concentrations, which is a technical limitation of the spectroscopy technique. Whilst the spectroscopy results were interesting, these were only analyzable on a subgroup (*N* ≤ 14) of this small study.

These factors combined result in a study which is likely to be statistically underpowered and therefore susceptible to under-or over-representing reported significant biomarker correlations. Because of these statistical limitations, we did not attempt to correct for multiple correlations which would have reduced the statistical significance of the correlations identified. However, we hope to have highlighted some biomarkers of potential clinical interest for future research (including a novel application of lower limb MRI biomarkers) in addition to identifying some key practical problems that could be addressed in the design of further studies. Specifically, we would welcome future studies which included a broader range of stroke severities, a larger sample size, and a narrower window of stroke onset times and hope we have demonstrated some of the practical barriers that would need to be overcome to enable such future research. Specifically, longitudinal observation of changes in the IMCL/EMCL ratio following stroke would be helpful given the lack of available literature and potentially relevant findings identified in our study. Furthermore, motor recovery is only one of a number of functions affected in patients with stroke, and we would welcome future research investigating MRI biomarkers, which may reflect recovery potential for speech or cognitive impairment too. As rehabilitation interventions for stroke grow in complexity, the need for biomarkers to both predict and measure recovery will grow—interventional studies may also present an opportunity to further MRI biomarker research in this field.

## 5. Conclusion

This study supports previous literature indicating a positive correlation between the integrity of the corticospinal tract and motor recovery post-stroke, expanding the limited available literature describing this relationship specifically for the lower limb. However, recovery of functional ambulation behaved differently to other clinical recovery markers by demonstrating an inverse relationship with corticospinal tract integrity.

We have also presented a novel protocol for the combined imaging of muscle biomarkers with brain imaging in stroke patients. We have demonstrated that this is possible in prospective observational research settings, although it does have technical limitations; particularly in the application of spectroscopy to assess microscopic fat distribution within muscles and transferability to all scanners in common clinical use.

Our study was exploratory in nature and therefore not statistically powered to enable reliable inferences; however, the correlations identified between both brain and leg MRI biomarkers and markers of clinical lower limb recovery may be of potential interest for future research.

## Data availability statement

The original contributions presented in the study are included in the article/Supplementary material, further inquiries can be directed to the corresponding author.

## Ethics statement

The studies involving humans were approved by North of Scotland Research Ethics Committee. The studies were conducted in accordance with the local legislation and institutional requirements. The participants provided their written informed consent to participate in this study.

## Author contributions

All authors contributed to the planning, conduct, and interpretation of the study results. All authors contributed to the article and approved the submitted version.

## Supplementary material

Appendix 1 Fugl-Meyer assessment.

Appendix 2 Functional ambulation category.

Appendix 3 Lower limb dynamometry.

Appendix 4 Motor NIHSS.

Appendix 5 MRI acquisition sequences and parameters.
