# Supplementary material for: A prospective study of MRI biomarkers in the brain and lower limb muscles for prediction of lower limb motor recovery following stroke
Source: Front Neurol. 2023 Oct 24;14:1229681. doi: 10.3389/fneur.2023.1229681 (PMC10628497; doi:10.3389/fneur.2023.1229681)
Supplement: Appendix 1 — Fugl-Meyer assessment. [file Table_1.DOCX]

1. Fugl-Meyer Assessment - Lower Extremity (FMA-LE)

Assessment of motor function (~10 minutes)

**Affected leg:** Left Right

***Please circle the patient’s scores for each sub-category. For item A, record the sub-totals and then add them together for the total score. For item B, please circle each of the patient’s scores and then add them together for each section total. Please then record the scores in the ‘Totals A-B’ box and sum them for the overall score.***

| **A) Lower Extremity – Need reflex hammer and stopwatch. Wash hands.** | | | | | | | |
| --- | --- | --- | --- | --- | --- | --- | --- |
| III. **Volitional movement mixing synergies**  (sitting position, knee 10cm away from edge of chair/bed). **Compare with unaffected side.** | | | | | **None** | **Partial** | **Full** |
| **Knee flexion** from actively or passively extended knee. Compare with unaffected | No active motion | | | | 0 |  |  |
|  | Less than 90° active flexion, palpate tendons of hamstrings | | | |  | 1 |  |
|  | More than 90° active flexion | | | |  |  | 2 |
| **Ankle dorsiflexion** compare with unaffected side | No active motion | | | | 0 |  |  |
|  | Limited dorsiflexion | | | |  | 1 |  |
|  | Complete dorsiflexion | | | |  |  | 2 |
| **Subtotal III** (max 4) | | | | | /4 | | |
| IV. **Volitional movement with little or no synergy** (standing position, hip 0°) | | | | | **None** | **Partial** | **Full** |
| **Knee flexion to 90°** hip at 0°. Lock and raise bed so patient can use headboard rail. Compare with unaffected side. | No active motion or immediate, simultaneous hip flexion | | | | 0 |  |  |
|  | Less than 90° knee flexion and/or hip flexion during movement | | | |  | 1 |  |
|  | At least 90° knee flexion without simultaneous hip flexion | | | |  |  | 2 |
| **Ankle dorsiflexion** compare with unaffected side. Balance support. | No active motion | | | | 0 |  |  |
|  | Limited dorsiflexion | | | |  | 1 |  |
|  | Complete dorsiflexion | | | |  |  | 2 |
| **Subtotal IV** (max 4) | | | | | /4 | | |
| I. **Reflex activity** (supine position). Muscles relaxed. Reflexes visualised or palpated. | | | | | **None** | **Can be elicited** | |
| **Flexors**: knee flexors of the **affected leg** (put knee under patient’s leg) | | | | | 0 | 2 | |
| **Extensors**: At least one: patellar (knee under patient leg), achilles (tilt & flex foot) **affected leg** | | | | | 0 | 2 | |
| **Subtotal I** (max 4) | | | | | /4 | | |
| V. **Normal reflex activity** (supine position, scored only if full score of 4 points is achieved in part IV. **Compare with the unaffected side**. | | | | | **0 (IV), Hyper** | **Lively** | **Normal** |
| **Reflex activity** knee flexors, Patellar, Achilles. | | 0 points on part IV or 2 of 3 reflexes markedly hyperactive | | | 0 |  |  |
|  |  | 1 reflex markedly hyperactive or at least 2 reflexes lively | | |  | 1 |  |
|  |  | Maximum of 1 reflex lively, none hyperactive | | |  |  | 2 |
| **Subtotal V** (max 2) | | | | | /2 | | |
| II. **Volitional movement within synergies** (supine position). **Compare with unaffected side.** | | | | | **None** | **Partial** | **Full** |
| **Flexor synergy**: Maximal hip flexion (abduction/external rotation), maximal flexion in knee and ankle joint (palpate distal tendons to ensure active knee flexion). | | | Hip | Flexion | 0 | 1 | 2 |
|  |  |  | Knee | Flexion | 0 | 1 | 2 |
|  |  |  | Ankle | Dorsiflexion | 0 | 1 | 2 |
| **Extensor synergy**: From flexor synergy to the hip extension/adduction, knee extension and ankle plantar flexion. Resistance is applied to ensure active movement, evaluate both movement and strength. | | | Hip | Extension | 0 | 1 | 2 |
|  |  |  |  | Adduction | 0 | 1 | 2 |
|  |  |  | Knee | Extension | 0 | 1 | 2 |
|  |  |  | Ankle | Plantar flexion | 0 | 1 | 2 |
| **Subtotal II** (max 14) | | | | | /14 | | |
| **Total A (max 28)** | | | | | /28 | | |

| **B)** **Co-ordination/Speed** (supine, after one trial with both legs, eyes closed, heel to knee cap of the opposite leg, 5 times as fast as possible (No movement = score 0). **Compare with unaffected side.** | | **Marked** | **Slight** | **None** |
| --- | --- | --- | --- | --- |
| **Tremor** (interference with co-ordination) | At least 1 completed movement | 0 | 1 | 2 |
| **Dysmetria** at least 1 completed movement (can ask patient if heel position expected) | Pronounced or unsystematic | 0 |  |  |
|  | Slight and systematic |  | 1 |  |
|  | No dysmetria |  |  | 2 |
|  |  | **≥ 6s** | **2-5s** | **< 2s** |
| **Time** start and end with the hand on the knee.  Use stopwatch and stop after >/=6 second difference. 0 if 5 movements incomplete. | At least 6 seconds slower than unaffected side | 0 |  |  |
|  | 2-5 seconds slower than unaffected side |  | 1 |  |
|  | Less than 2 seconds difference |  |  | 2 |
| **Total B (max 6)** | | /6 | | |

Left leg speed: Right leg speed:

| Totals A-B | |
| --- | --- |
| A) Lower Extremity | /28 |
| B) Co-ordination/Speed | /6 |
| Total A-B (motor function) | /34 |
