# Supplementary material for: A prospective study of MRI biomarkers in the brain and lower limb muscles for prediction of lower limb motor recovery following stroke
Source: Front Neurol. 2023 Oct 24;14:1229681. doi: 10.3389/fneur.2023.1229681 (PMC10628497; doi:10.3389/fneur.2023.1229681)
Supplement: Appendix 2 — Functional ambulation category. [file Table_2.DOCX]

Functional Ambulation Category (FAC) (2 minutes)

***Please choose only one description which best describes the patient’s walking ability (regardless of use of walking aids) and record the score in the box provided. This can be scored based on walking ability already demonstrated by the patient. Ask if they can climb stairs independently and take them upstairs (carefully) if they are unsure.***

| **Score** | **Category** | **Description** |
| --- | --- | --- |
| 0 | Non-functional ambulator | Patient cannot walk, or needs help from 2 or more persons. |
| 1 | Ambulator, dependent on physical assistance – level I | Patient requires continuous manual contact to support body weight as well as to maintain balance or to assist coordination. |
| 2 | Ambulator, dependent on physical assistance – level II | Patient requires intermittent or continuous light touch to assist balance or coordination. |
| 3 | Ambulator, dependent on supervision | Patient can ambulate on level surface without manual contact of another person but requires standby guarding of one person either for safety or verbal cueing. |
| 4 | Ambulator, independent level surface only | Patient can ambulate independently on level surface but requires supervision to negotiate (e.g. stairs, inclines, non-level surfaces). |
| 5 | Ambulator, independent | Patient can walk everywhere independently, including stairs. |
| **Score:** |  |  |
