# Supplementary material for: A prospective study of MRI biomarkers in the brain and lower limb muscles for prediction of lower limb motor recovery following stroke
Source: Front Neurol. 2023 Oct 24;14:1229681. doi: 10.3389/fneur.2023.1229681 (PMC10628497; doi:10.3389/fneur.2023.1229681)
Supplement: Appendix 3 — Lower limb dynamometry. [file Table_3.DOCX]

Lower Limb Dynamometry (~15 minutes)

| [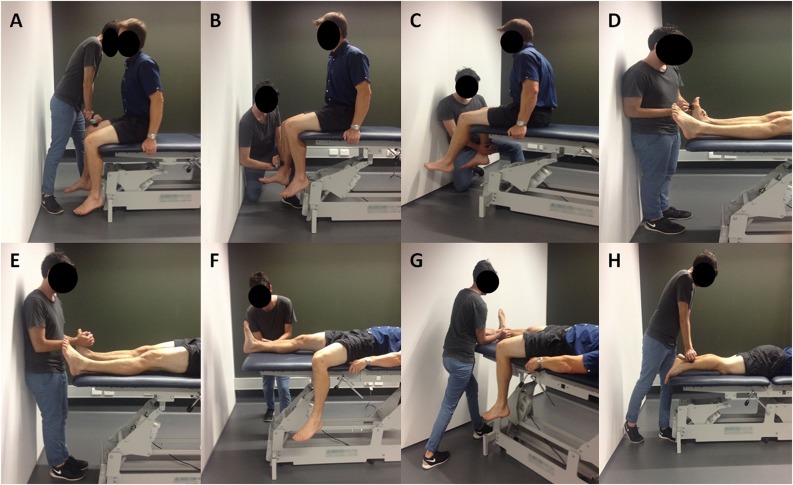](https://www.ncbi.nlm.nih.gov/core/lw/2.0/html/tileshop_pmc/tileshop_pmc_inline.html?title=Click%20on%20image%20to%20zoom&p=PMC3&id=4624940_pone.0140822.g001.jpg) | | [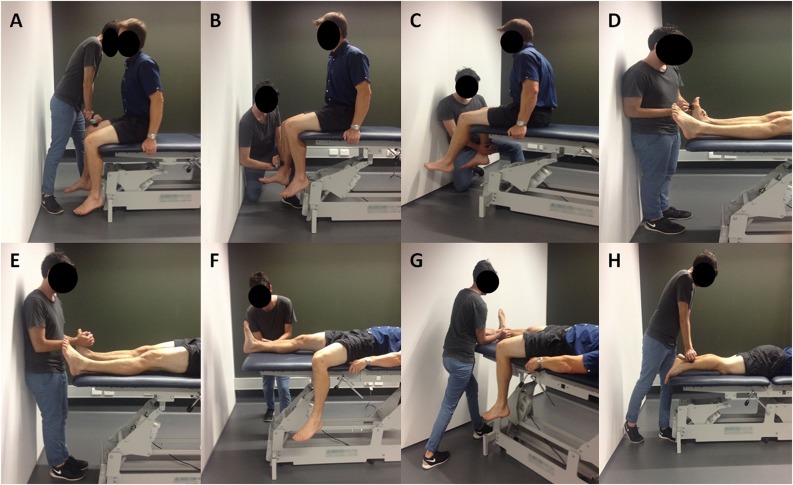](https://www.ncbi.nlm.nih.gov/core/lw/2.0/html/tileshop_pmc/tileshop_pmc_inline.html?title=Click%20on%20image%20to%20zoom&p=PMC3&id=4624940_pone.0140822.g001.jpg) | | [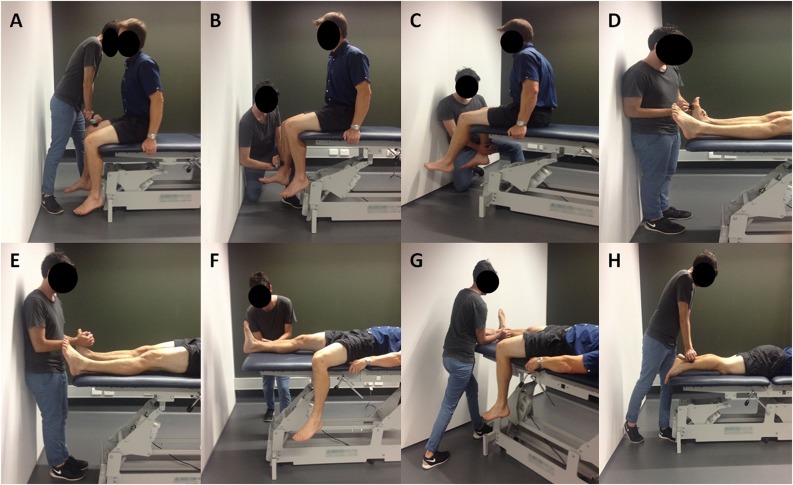](https://www.ncbi.nlm.nih.gov/core/lw/2.0/html/tileshop_pmc/tileshop_pmc_inline.html?title=Click%20on%20image%20to%20zoom&p=PMC3&id=4624940_pone.0140822.g001.jpg) | | [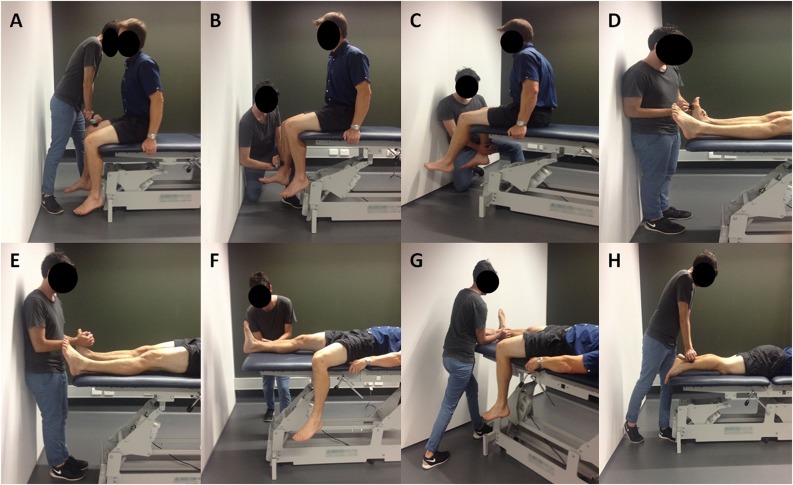](https://www.ncbi.nlm.nih.gov/core/lw/2.0/html/tileshop_pmc/tileshop_pmc_inline.html?title=Click%20on%20image%20to%20zoom&p=PMC3&id=4624940_pone.0140822.g001.jpg) | |
| --- | --- | --- | --- | --- | --- | --- | --- |
| D) **Ankle plantarflexors**  Participant lying supine with the ankle in plantargrade and hips and knees extended. Dynamometer placed over the metatarsal heads on the sole of the foot. Round dyno head. | | E) **Ankle dorsiflexors** Participant lying supine with the ankle relaxed and hips and knees extended.  Dynamometer placed over the metatarsal heads on the dorsum of the foot. Round dyno head. | | F) **Hip abductors** Participant lying supine and hips and knees extended.  Dynamometer placed on the lateral aspect of the shank, proximal to the ankle joint. Round dyno head. | | G) **Hip adductors** Participant lying supine and hips and knees extended. Dynamometer placed on the medial aspect of the shank, proximal to the ankle joint.  Round dyno head. | |
| A) Right leg: | | B) Right leg: | | C) Right leg: | | D) Right leg: | |
| 1. | \|  \|  \| . \|  \|  \| Kg \| \| --- \| --- \| --- \| --- \| --- \| --- \| | 1. | \|  \|  \| . \|  \|  \| Kg \| \| --- \| --- \| --- \| --- \| --- \| --- \| | 1. | \|  \|  \| . \|  \|  \| Kg \| \| --- \| --- \| --- \| --- \| --- \| --- \| | 1. | \|  \|  \| . \|  \|  \| Kg \| \| --- \| --- \| --- \| --- \| --- \| --- \| |
| 2. | \|  \|  \| . \|  \|  \| Kg \| \| --- \| --- \| --- \| --- \| --- \| --- \| | 2. | \|  \|  \| . \|  \|  \| Kg \| \| --- \| --- \| --- \| --- \| --- \| --- \| | 2. | \|  \|  \| . \|  \|  \| Kg \| \| --- \| --- \| --- \| --- \| --- \| --- \| | 2. | \|  \|  \| . \|  \|  \| Kg \| \| --- \| --- \| --- \| --- \| --- \| --- \| |
| 3. | \|  \|  \| . \|  \|  \| Kg \| \| --- \| --- \| --- \| --- \| --- \| --- \| | 3. | \|  \|  \| . \|  \|  \| Kg \| \| --- \| --- \| --- \| --- \| --- \| --- \| | 3. | \|  \|  \| . \|  \|  \| Kg \| \| --- \| --- \| --- \| --- \| --- \| --- \| | 3. | \|  \|  \| . \|  \|  \| Kg \| \| --- \| --- \| --- \| --- \| --- \| --- \| |
| Av | \|  \|  \| . \|  \|  \| Kg \| \| --- \| --- \| --- \| --- \| --- \| --- \| | Av | \|  \|  \| . \|  \|  \| Kg \| \| --- \| --- \| --- \| --- \| --- \| --- \| | Av | \|  \|  \| . \|  \|  \| Kg \| \| --- \| --- \| --- \| --- \| --- \| --- \| | Av | \|  \|  \| . \|  \|  \| Kg \| \| --- \| --- \| --- \| --- \| --- \| --- \| |
| A) Left leg: | | B) Left leg: | | C) Left leg: | | D) Left leg: | |
| 1. | \|  \|  \| . \|  \|  \| Kg \| \| --- \| --- \| --- \| --- \| --- \| --- \| | 1. | \|  \|  \| . \|  \|  \| Kg \| \| --- \| --- \| --- \| --- \| --- \| --- \| | 1. | \|  \|  \| . \|  \|  \| Kg \| \| --- \| --- \| --- \| --- \| --- \| --- \| | 1. | \|  \|  \| . \|  \|  \| Kg \| \| --- \| --- \| --- \| --- \| --- \| --- \| |
| 2. | \|  \|  \| . \|  \|  \| Kg \| \| --- \| --- \| --- \| --- \| --- \| --- \| | 2. | \|  \|  \| . \|  \|  \| Kg \| \| --- \| --- \| --- \| --- \| --- \| --- \| | 2. | \|  \|  \| . \|  \|  \| Kg \| \| --- \| --- \| --- \| --- \| --- \| --- \| | 2. | \|  \|  \| . \|  \|  \| Kg \| \| --- \| --- \| --- \| --- \| --- \| --- \| |
| 3. | \|  \|  \| . \|  \|  \| Kg \| \| --- \| --- \| --- \| --- \| --- \| --- \| | 3. | \|  \|  \| . \|  \|  \| Kg \| \| --- \| --- \| --- \| --- \| --- \| --- \| | 3. | \|  \|  \| . \|  \|  \| Kg \| \| --- \| --- \| --- \| --- \| --- \| --- \| | 3. | \|  \|  \| . \|  \|  \| Kg \| \| --- \| --- \| --- \| --- \| --- \| --- \| |
| Av | \|  \|  \| . \|  \|  \| Kg \| \| --- \| --- \| --- \| --- \| --- \| --- \| | Av | \|  \|  \| . \|  \|  \| Kg \| \| --- \| --- \| --- \| --- \| --- \| --- \| | Av | \|  \|  \| . \|  \|  \| Kg \| \| --- \| --- \| --- \| --- \| --- \| --- \| | Av | \|  \|  \| . \|  \|  \| Kg \| \| --- \| --- \| --- \| --- \| --- \| --- \| |
|  |  |  |  |  |  |  |  |
| [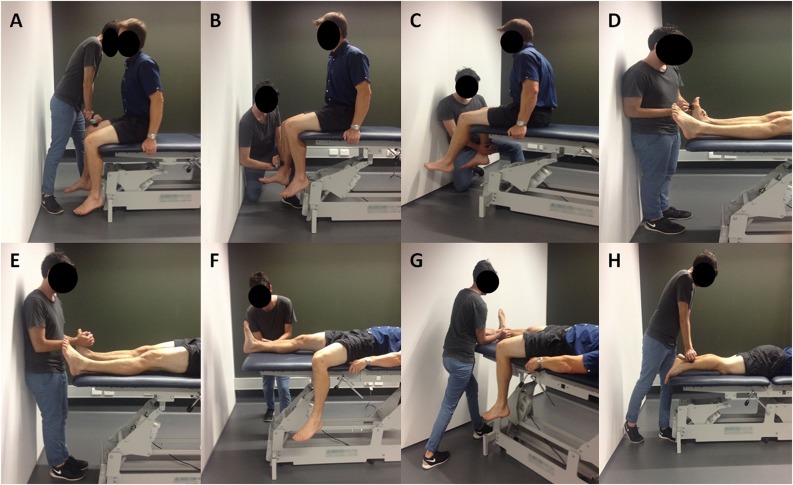](https://www.ncbi.nlm.nih.gov/core/lw/2.0/html/tileshop_pmc/tileshop_pmc_inline.html?title=Click%20on%20image%20to%20zoom&p=PMC3&id=4624940_pone.0140822.g001.jpg) | | [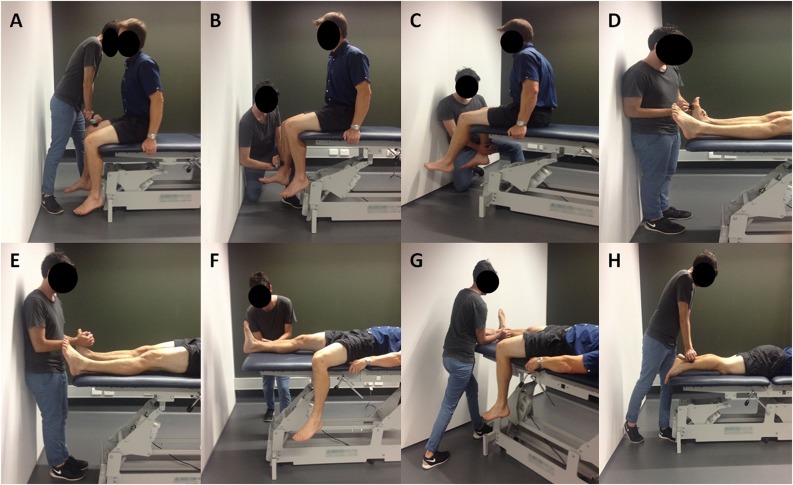](https://www.ncbi.nlm.nih.gov/core/lw/2.0/html/tileshop_pmc/tileshop_pmc_inline.html?title=Click%20on%20image%20to%20zoom&p=PMC3&id=4624940_pone.0140822.g001.jpg) | | [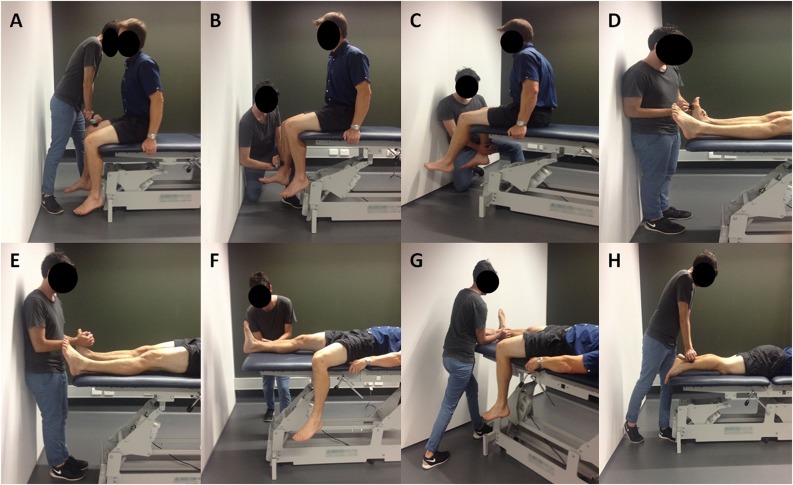](https://www.ncbi.nlm.nih.gov/core/lw/2.0/html/tileshop_pmc/tileshop_pmc_inline.html?title=Click%20on%20image%20to%20zoom&p=PMC3&id=4624940_pone.0140822.g001.jpg) | | [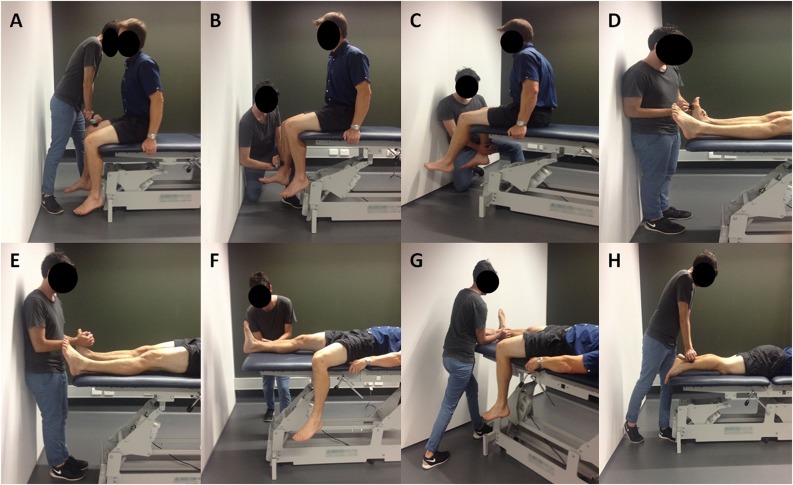](https://www.ncbi.nlm.nih.gov/core/lw/2.0/html/tileshop_pmc/tileshop_pmc_inline.html?title=Click%20on%20image%20to%20zoom&p=PMC3&id=4624940_pone.0140822.g001.jpg) | |
| H) **Hip extensors**  Participant lying prone and hips and knees extended. Dynamometer placed on the posterior aspect of the shank, proximal to the ankle joint. Curved dyno head.  **ENSURE PARTICIPANT DOES NOT BEND KNEE** | | A) **Hip flexors** Participant seated and hips and knees flexed at 90°.  Dynamometer placed on the anterior aspect of the thigh, proximal to the knee joint.  Patient’s feet shouldn’t touch the floor. Arms crossed if possible. Curved dyno head. | | B) **Knee extensors** Participant seated and hip and knees flexed at 90°.  Dynamometer placed on the anterior aspect of the shank, proximal to the ankle joint.  Patient’s feet shouldn’t touch the floor. Arms crossed if possible. Curved dyno head. | | C) **Knee flexors**  Participant seated and hips and knees flexed at 90°. Dynamometer placed on the posterior aspect of the shank, proximal to the ankle joint. Patient’s feet shouldn’t touch the floor. Arms crossed if possible. Curved dyno head. | |
| E) Right leg: | | F) Right leg: | | G) Right leg: | | H) Right leg: | |
| 1. | \|  \|  \| . \|  \|  \| Kg \| \| --- \| --- \| --- \| --- \| --- \| --- \| | 1. | \|  \|  \| . \|  \|  \| Kg \| \| --- \| --- \| --- \| --- \| --- \| --- \| | 1. | \|  \|  \| . \|  \|  \| Kg \| \| --- \| --- \| --- \| --- \| --- \| --- \| | 1. | \|  \|  \| . \|  \|  \| Kg \| \| --- \| --- \| --- \| --- \| --- \| --- \| |
| 2. | \|  \|  \| . \|  \|  \| Kg \| \| --- \| --- \| --- \| --- \| --- \| --- \| | 2. | \|  \|  \| . \|  \|  \| Kg \| \| --- \| --- \| --- \| --- \| --- \| --- \| | 2. | \|  \|  \| . \|  \|  \| Kg \| \| --- \| --- \| --- \| --- \| --- \| --- \| | 2. | \|  \|  \| . \|  \|  \| Kg \| \| --- \| --- \| --- \| --- \| --- \| --- \| |
| 3. | \|  \|  \| . \|  \|  \| Kg \| \| --- \| --- \| --- \| --- \| --- \| --- \| | 3. | \|  \|  \| . \|  \|  \| Kg \| \| --- \| --- \| --- \| --- \| --- \| --- \| | 3. | \|  \|  \| . \|  \|  \| Kg \| \| --- \| --- \| --- \| --- \| --- \| --- \| | 3. | \|  \|  \| . \|  \|  \| Kg \| \| --- \| --- \| --- \| --- \| --- \| --- \| |
| Av | \|  \|  \| . \|  \|  \| Kg \| \| --- \| --- \| --- \| --- \| --- \| --- \| | Av | \|  \|  \| . \|  \|  \| Kg \| \| --- \| --- \| --- \| --- \| --- \| --- \| | Av | \|  \|  \| . \|  \|  \| Kg \| \| --- \| --- \| --- \| --- \| --- \| --- \| | Av | \|  \|  \| . \|  \|  \| Kg \| \| --- \| --- \| --- \| --- \| --- \| --- \| |
| E) Left leg: | | F) Left leg: | | G) Left leg: | | H) Left leg: | |
| 1. | \|  \|  \| . \|  \|  \| Kg \| \| --- \| --- \| --- \| --- \| --- \| --- \| | 1. | \|  \|  \| . \|  \|  \| Kg \| \| --- \| --- \| --- \| --- \| --- \| --- \| | 1. | \|  \|  \| . \|  \|  \| Kg \| \| --- \| --- \| --- \| --- \| --- \| --- \| | 1. | \|  \|  \| . \|  \|  \| Kg \| \| --- \| --- \| --- \| --- \| --- \| --- \| |
| 2. | \|  \|  \| . \|  \|  \| Kg \| \| --- \| --- \| --- \| --- \| --- \| --- \| | 2. | \|  \|  \| . \|  \|  \| Kg \| \| --- \| --- \| --- \| --- \| --- \| --- \| | 2. | \|  \|  \| . \|  \|  \| Kg \| \| --- \| --- \| --- \| --- \| --- \| --- \| | 2. | \|  \|  \| . \|  \|  \| Kg \| \| --- \| --- \| --- \| --- \| --- \| --- \| |
| 3. | \|  \|  \| . \|  \|  \| Kg \| \| --- \| --- \| --- \| --- \| --- \| --- \| | 3. | \|  \|  \| . \|  \|  \| Kg \| \| --- \| --- \| --- \| --- \| --- \| --- \| | 3. | \|  \|  \| . \|  \|  \| Kg \| \| --- \| --- \| --- \| --- \| --- \| --- \| | 3. | \|  \|  \| . \|  \|  \| Kg \| \| --- \| --- \| --- \| --- \| --- \| --- \| |
| Av | \|  \|  \| . \|  \|  \| Kg \| \| --- \| --- \| --- \| --- \| --- \| --- \| | Av | \|  \|  \| . \|  \|  \| Kg \| \| --- \| --- \| --- \| --- \| --- \| --- \| | Av | \|  \|  \| . \|  \|  \| Kg \| \| --- \| --- \| --- \| --- \| --- \| --- \| | Av | \|  \|  \| . \|  \|  \| Kg \| \| --- \| --- \| --- \| --- \| --- \| --- \| |
