# Supplementary material for: A prospective study of MRI biomarkers in the brain and lower limb muscles for prediction of lower limb motor recovery following stroke
Source: Front Neurol. 2023 Oct 24;14:1229681. doi: 10.3389/fneur.2023.1229681 (PMC10628497; doi:10.3389/fneur.2023.1229681)
Supplement: Appendix 4 — Motor NIHSS. [file Table_4.DOCX]

Current Lower Limb Motor National Institutes of Health Stroke Scale (NIHSS) (~5 minutes)

The limb is placed in the appropriate position: hold the leg at 30 degrees (always tested supine). Drift is scored if the leg falls before 5 seconds. Each limb is tested in turn, beginning with the non-paretic leg. Only in the case of amputation or joint fusion at the hip, the examiner should record the score as untestable (UN) and record the reason for this.

| Motor function leg | 0 | Normal (holds leg for 5 sec without drift) | **Right**  Affected leg |  |
| --- | --- | --- | --- | --- |
|  | 1 | Drift |  |  |
|  | 2 | Some effort against gravity |  |  |
|  | 3 | No effort against gravity | **Left**  Affected leg |  |
|  | 4 | No movement |  |  |
|  |  | Untestable (limb amputated) |  |  |
