# Supplementary material for: A prospective study of MRI biomarkers in the brain and lower limb muscles for prediction of lower limb motor recovery following stroke
Source: Front Neurol. 2023 Oct 24;14:1229681. doi: 10.3389/fneur.2023.1229681 (PMC10628497; doi:10.3389/fneur.2023.1229681)
Supplement: Appendix 5 — MRI acquisition sequences and parameters. [file Table_5.DOCX]

Appendix 5 – MRI acquisition sequences and paramaters

Leg imaging acquisition

Structural imaging was performed with a T1-weighted Turbo Spin Echo sequence (TR/TE = 650ms/10ms) acquired and reconstructed at 1.36mm x 1.36mm with 5mm contiguous slices (~5.5 mins). Short Tau Inversion Recovery imaging was acquired to assess macroscopic oedema (TR/TI/TE = 4000ms/220ms/37ms) at 1.00mm x 1.00mm and reconstructed to 0.57mm x 0.57mm with 10.00mm slices and additional 10mm interslice gap (~5.8 mins). Quantitative fat-fraction maps were calculated using 3-point mDixon (Philips) with echoes at –π, 0, and + π (ΔTE = 1.15ms, TR/TE = 100ms/3.45ms) acquired at 1.00mm x 1.00mm and reconstructed to 0.57mm x 0.57mm with 10.00mm slices with additional 10mm interslice gap (~6 mins). Point RESolved Spectroscopy (PRESS) was performed at rest with voxels placed in Vastus Lateralis bilaterally (30mm x 30mm x 60mm each, TR/TE = 2000ms/50ms) to extract intra- and extra-myocellular lipid concentrations (IMCL and EMCL), absolute and normalised to creatine (~10 mins).

Brain imaging acquisition

Whole-brain structural imaging was acquiring using a 3D Magnetisation Prepared Rapid Acquisition with Gradient Echo (MPRAGE) T1-weighted sagittally orientated volumetric sequence (TR/TE = 8.3ms/4.6ms, flip-angle = 8°, SENSE factor = 2) acquired and reconstructed to 1.00mm x 1.00mm x 1.00mm (~4.4 mins). An anisotropic structural T2-weighted Turbo Spin Echo sequence was also performed (TR/TE = 3000ms/80ms) acquired at 0.55mm x 0.65mm and reconstructed to 0.40mm x 0.40mm with 3mm contiguous slices (~3 mins). Diffusion tensor imaging was acquired using a 2D Spin-Echo, single-shot Echo Planar sequence acquired at 2.20mm x 2.25mm and reconstructed to 2.11mm x 2.11mm with 2.5mm contiguous slices. Diffusion-weighting was applied in 64 uniformly distributed directions (b=1000 s.mm^-2^) including 6 acquisitions with no diffusion weighting (b=0 s.mm^-2^), and an identical non-diffusion weighted image was acquired with reversed phase encoding gradients for distortion correction (~7.3 mins).

Automatic tractography analysis

Automatic CST analysis was performed with manual supervision in DSI Studio^1–4^.

The diffusion MRI data were rotated to align with the AC-PC line. The accuracy of b-table orientation was examined by comparing fiber orientations with those of a population-averaged template^4^. The tensor metrics were calculated using DWI with b-value lower than 1750 s/mm². A deterministic fiber tracking algorithm^3^ was used with augmented tracking strategies^1^ to improve reproducibility. The anatomy prior of a tractography atlas^4^ was used to map the corticospinal tracts with a distance tolerance of 16mm in the ICBM152 space. A seeding region was placed in both corticospinal tracts. The anisotropy threshold was randomly selected. The angular threshold was randomly selected from 15 degrees to 90 degrees. The step size was randomly selected from 0.5 voxel to 1.5 voxels. Tracks with length shorter than 10mm or longer than 200mm were discarded. A total of 1000000 seeds were placed. Topology-informed pruning^2^ was applied to the tractography with 16 iteration(s) to remove false connections. The whole tract volume and average FA were extracted on either side.

Image processing and reconstruction

Spectroscopy data were exported in PAR/REC format. All other imaging data were exported from the scanner in both conventional and enhanced DICOM formats. The Dixon images were converted to ‘Analyze’ format for quantitative fat-mapping utilising dcm2niix, but the remaining leg images were analysed in DICOM format. All brain imaging was converted to NIfTI-1 format and distortion correction was performing using TOP-UP/EDDY algorithms from the FSL software package.

Bibliography

1. Yeh FC. Shape analysis of the human association pathways. *Neuroimage*. 2020;223:117329. doi:10.1016/J.NEUROIMAGE.2020.117329

2. Yeh FC, Panesar S, Barrios J, et al. Automatic Removal of False Connections in Diffusion MRI Tractography Using Topology-Informed Pruning (TIP). *Neurotherapeutics*. 2019;16(1):52. doi:10.1007/S13311-018-0663-Y

3. Yeh FC, Verstynen TD, Wang Y, Fernández-Miranda JC, Tseng WY. Deterministic Diffusion Fiber Tracking Improved by Quantitative Anisotropy. *PLoS One*. 2013;8(11):80713. doi:10.1371/journal.pone.0080713

4. Yeh FC, Panesar S, Fernandes D, et al. Population-averaged atlas of the macroscale human structural connectome and its network topology. *Neuroimage*. 2018;178:57-68. doi:10.1016/J.NEUROIMAGE.2018.05.027
